# Improving Bone Health Care and Monitoring of Intellectual Disability Patients on a Low Secure Female Unit

**DOI:** 10.1192/bjo.2024.380

**Published:** 2024-08-01

**Authors:** Serena Hinze, Rosie England, Ambreen Rashid, Nicole Freeman

**Affiliations:** 1 Coventry and Warwickshire Partnership NHS Trust, Coventry, United Kingdom; 2 Black Country Healthcare NHS Foundation Trust, Walsall, United Kingdom

## Abstract

**Aims:**

- To ensure all patients on a low secure female inpatient unit have bone health risk factors assessed, identified and interventions initiated within 3 months of admission.
- The above to be achieved through creation of a Bone Health proforma, integration of a Bone Health checklist into the Intellectual Disability (ID) Annual Health Check and delivery of bone health education for patients and staff.

Background

Intellectual Disability has been shown to be associated with poor bone health, osteoporosis and increased fracture risk. The current NICE guidelines and risk tools (QFRACTURE), do not adequately reflect the true risk within this patient group who present with additional risks of epilepsy, antiepileptic medication and greater likelihood of low vitamin D. Bone health has not routinely been monitored in this population hitherto. This quality improvement project sought to develop a process whereby potential risk factors for poor bone health were identified and managed effectively.

**Methods:**

The project was undertaken between February 2022 – October 2023 on a female low secure unit. All 8 patients on the unit were included. A baseline screening of risk factors was conducted to assess current practice and explore the clinical need for the project. Most patients were found to have multiple risk factors which had not previously been highlighted, indicating the need for formalised monitoring. Based on questionnaire feedback, a Bone Health Care Plan, a risk factor checklist which was integrated with patients’ ID Annual Health check and Educational workshops were developed. Primary and secondary drivers were identified at the outset and plan, do, study, act cycles were used to refine change ideas. The changes were evaluated using quantitative and qualitative measures.

**Results:**

Every inpatient has a completed Bone Health Care Plan. Twenty-five percent of patients were identified as having a particularly high risk and have had referrals accepted for Dual-energy X-ray absorptiometry (DEXA) scans. All patients are using a new easy-read ID Annual Health Check form with Bone Health checklist incorporated. All staff and patients were given the opportunity to attend a series of four bone health workshops, 43% of patients attended at least one session. Positive written and verbal feedback was received from both patients and staff.

**Conclusion:**

100% of service users have had their risk factors for bone health assessed and any necessary interventions applied. There is now an embedded process for reviewing the bone health of these patients annually where previously there was no regular monitoring.

